# Global dissemination of H5N1 influenza viruses bearing the clade 2.3.4.4b HA gene and biologic analysis of the ones detected in China

**DOI:** 10.1080/22221751.2022.2088407

**Published:** 2022-06-28

**Authors:** Pengfei Cui, Jianzhong Shi, Congcong Wang, Yuancheng Zhang, Xin Xing, Huihui Kong, Cheng Yan, Xianying Zeng, Liling Liu, Guobin Tian, Chengjun Li, Guohua Deng, Hualan Chen

**Affiliations:** a State Key Laboratory of Veterinary Biotechnology, Harbin Veterinary Research Institute, CAAS, Harbin, People’s Republic of China; b National Poultry Laboratory Animal Resource Center, Harbin Veterinary Research Institute, CAAS, Harbin, People’s Republic of China

**Keywords:** Avian influenza virus, H5N1, clade2.3.4.4b, evolution, pathogenicity, antigenicity

## Abstract

H5N1 avian influenza viruses bearing the clade 2.3.4.4b hemagglutinin gene have been widely circulating in wild birds and are responsible for the loss of over 70 million domestic poultry in Europe, Africa, Asia, and North America since October 2020. During our routine surveillance, 13 H5N1 viruses were isolated from 26,767 wild bird and poultry samples that were collected between September 2021 and March 2022 in China. To investigate the origin of these Chinese isolates and understand their genetic relationship with the globally circulating H5N1 viruses, we performed a detailed phylogenic analysis of 233 representative H5N1 strains that were isolated from 28 countries. We found that, after they emerged in the Netherlands, the H5N1 viruses encountered complicated gene exchange with different viruses circulating in wild birds and formed 16 genotypes. Genotype one (G1) was predominant, being detected in 22 countries, whereas all other genotypes were only detected in one or two continents. H5N1 viruses of four genotypes (G1, G7, G9, and G10) were detected in China; three of these genotypes have been previously reported in other countries. The H5N1 viruses detected in China replicated in mice, with pathogenicity varying among strains; the G1 virus was highly lethal in mice. Moreover, we found that these viruses were antigenically similar to and well matched with the H5-Re14 vaccine strain currently used in China. Our study reveals the overall picture of H5N1 virus evolution and provides insights for the control of these viruses.

## Introduction

The H5 subtype influenza virus is the most widely detected avian influenza virus and has caused numerous disease outbreaks in domestic poultry and wild birds around the world [1]. After long-term circulation in nature, H5 viruses have undergone extensive evolution by accumulating mutations and reassorting with other influenza virus subtypes. The hemagglutinin (HA) gene of the H5 viruses detected in the last two decades has been categorized into different phylogenetic clades (clade 0 to clade 9) and different levels of subclade [2]. H5 viruses bearing the HA gene of most clades have been detected in limited geographic areas and have been eradicated by active control strategies applied in domestic poultry, including culling and vaccination. Only the strains bearing the clade 2.2 HA gene, clade 2.3.2 HA gene, or clade 2.3.4.4 HA gene have infected and been maintained in wild birds, which has led to their spread to multiple countries and continents [3–6].

H5 viruses that bearing the clade 2.3.4.4 HA gene have been detected in many countries over several continents since 2014 [7,8]. The clade 2.3.4.4 HA of H5 viruses has been further divided into eight subclades, namely clades 2.3.4.4a to 2.3.4.4h [3,9]. During the last three years, the H5 viruses bearing the clade 2.3.4.4b HA gene have attracted wide attention. At the beginning of 2020, H5N8 viruses bearing the clade 2.3.4.4b HA gene infected domestic poultry and wild birds, leading to the loss of over 33 million domestic birds in Europe, Africa, and Asia [10–13]. Moreover, these H5N8 viruses reassorted with other avian influenza viruses and formed H5N1, H5N2, H5N3, H5N4, H5N5, and H5N6 viruses [11,14,15]. Unlike the H5N2, H5N3, H5N4, H5N5, and H5N6 viruses, each of which has only been detected in countries of one or two continents, the H5N1 viruses bearing the clade 2.3.4.4b HA gene have spread to many countries in Europe, Africa, Asia, and America since they emerged in October 2020 in the Netherlands [16–19]. During our routine surveillance of poultry in China, H5N1 viruses bearing the HA of clade 2.3.4.4b were detected in wild birds and domestic poultry between September 2021 and March 2022, indicating that, like the H5N8 viruses [10,20], the H5N1 viruses have been introduced into China.

Influenza viruses easily exchange gene segments when different strains co-infect the same host, and the resulting strains with different gene constellations may have different biologic properties [21]. Although the H5N1 viruses bearing the clade 2.3.4.4b HA gene are widely detected on different continents, the genetic relationship of the strains detected in different countries is largely unknown, and their biologic properties are rarely evaluated. In this study, we performed a detail genetic analysis of 233 H5N1 representative viruses detected in different countries since October 2020, and found that these viruses formed 16 different genotypes by exchanging their NA and internal genes with other viruses circulating in wild birds. We also revealed the spatiotemporal spread of virus in each genotype. Moreover, we evaluated the pathogenicity in mice and antigenicity of the H5N1 viruses detected in China. Our study provides important information about the evolution and dissemination of different H5N1 viruses bearing the clade 2.3.4.4b HA gene, and provides insights for the control of these viruses.

## Materials and methods

### Ethics statements and facility

Swab samples collected during surveillance were processed in the enhanced biosafety level 2 (BSL2+) facility in the Harbin Veterinary Research Institute of the Chinese Academy of Agricultural Sciences (HVRI, CAAS). All experiments with live H5N1 viruses or organs collected from dead birds were carried out in the animal biosafety level 3 (ABSL3) facility in the HVRI. The study was carried out in strict accordance with the recommendations in the Guide for the Care and Use of Laboratory Animals of the Ministry of Science and Technology of China. The protocol for the animal studies was approved by the Committee on the Ethics of Animal Experiments of the HVRI, CAAS.

### H5 avian influenza epidemiology information

The epidemiology data for H5 highly pathogenic avian influenza from January 1, 2020 to April 1, 2022, were downloaded from EMPRES-i + (https://empres-i.apps.fao.org/epidemiology).

### Virus isolation

Swab samples (oropharyngeal swab and cloacal swab of the same bird were put in the same sample collection tube and counted as one sample) or organs from dead birds were individual inoculated into 10-day-old embryonated chicken eggs and incubated for 48 h at 37°C. The HA subtype was identified by using the hemagglutinin inhibition (HI) test, and the NA subtype was confirmed by direct sequence analysis. The H5N1 viruses were biologically cloned three times by limiting dilution in embryonated specific-pathogen-free (SPF) chicken eggs, and the virus stocks were grown in SPF chicken eggs and maintained at −70°C.

### Genetic and phylogenetic analyses

The genome of the H5N1 viruses was sequenced on an Applied Biosystems DNA Analyzer (3500xL Genetic Analyzer, USA). The nucleotide sequence was edited by using the Seqman module of the DNAStar package. The HA gene tree was generated with the HA of 263 H5 viruses bearing clade 2.3.4.4b HA, including 250 viruses (eight H5N6 human viruses, 22 H5N8 representative viruses, and 220 H5N1 representative viruses) reported previously by us and others [10,15,16,18,22–25] and 13 H5N1 viruses detected in China in this study. Phylogeographic analysis was performed using an asymmetric continuous-time Markov chain with Bayesian stochastic search variable selection implemented in BEAST (v1.10.4) [26]. A preliminary check using TempEst (v1.5.1) [27] confirmed a significant temporal signal in our dataset, which was proved by linear regression of root-to-tip distance against sampling date (R^2^ = 0.7027, correlation coefficient = 0.8383). The GTR+F+G4 substitution model, which was selected using the Bayesian information criterion by ModelFinder in IQ-TREE [28], was used along with an uncorrelated lognormal relaxed molecular clock and a Bayesian skygrid coalescent tree prior. Markov Chain Monte Carlo (MCMC) chains were run for 100 million iterations and sampled every 10000 steps to make sure that all parameters converged (Effective sample size values greater than 200), and the first 10% of samples were discarded as burn-in. The maximum clade credibility (MCC) tree was generated and summarized by TreeAnnotator (v1.10.4). Then, the ggtree package in R was used to visualize and annotate the MCC tree [29]. The trees of the NA and six internal genes were generated by the neighbor-joining method using the MEGA 7.0.14 software package. The tree topology was evaluated by 1,000 bootstrap analyses. The NA and six internal genes of representative influenza viruses were downloaded from the GISAID database, and 96% sequence identity cutoffs were used to categorize the groups of these genes in the phylogenetic trees.

### Replication and virulence of H5N1 viruses in mice

Groups of three 6-week-old female BALB/c mice (Beijing Experimental Animal Center, Beijing, China) were lightly anesthetized with CO_2_ and then inoculated intranasally (i.n.) with 10^6^ EID_50_ of the H5N1 virus in a volume of 50 μl, and their organs, including nasal turbinate, lungs, spleen, kidneys, and brain were collected on Day 3 post-inoculation (p.i.) for virus titration in chicken eggs. To assess the 50% mouse lethal dose (MLD_50_), groups of five mice were inoculated with 10-fold serial dilutions of the test virus containing 10^1^–10^6^ EID_50_, and body weight loss and mortality were monitored for 14 days.

### Antigenic analysis

Antigenic analysis of the viruses was performed by using the HI assay with 1.0% chicken erythrocytes. The chicken antisera used in this study were generated in 6-week-old SPF chickens. Briefly, chickens were inoculated with 0.5 mL of oil-emulsified inactivated vaccine seed virus, and serum was collected three weeks after vaccination. The vaccine seed viruses H5-Re11, H5-Re13, and H5-Re14 were generated by reverse genetics [30,31]; their surface genes are derived from A/duck/Guizhou/S4184/2017(H5N6) (clade 2.3.4.4h HA gene), A/duck/Fujian/S1424/2020(H5N6) (clade 2.3.4.4h HA gene), and A/whooper swan/Shanxi/4-1/2020(H5N8) (clade 2.3.4.4b HA gene), respectively, and their internal genes are from the A/Puerto Rico/8/1934 (H1N1) (PR8) virus [30,31]. Antigenic maps of the viruses were obtained by using Antigenic Cartography software (http://www.antigenic-cartography.org/).

## Results

### Avian influenza outbreaks caused by clade 2.3.4.4 HA-bearing H5 viruses since January 2020

**Figure 1.**
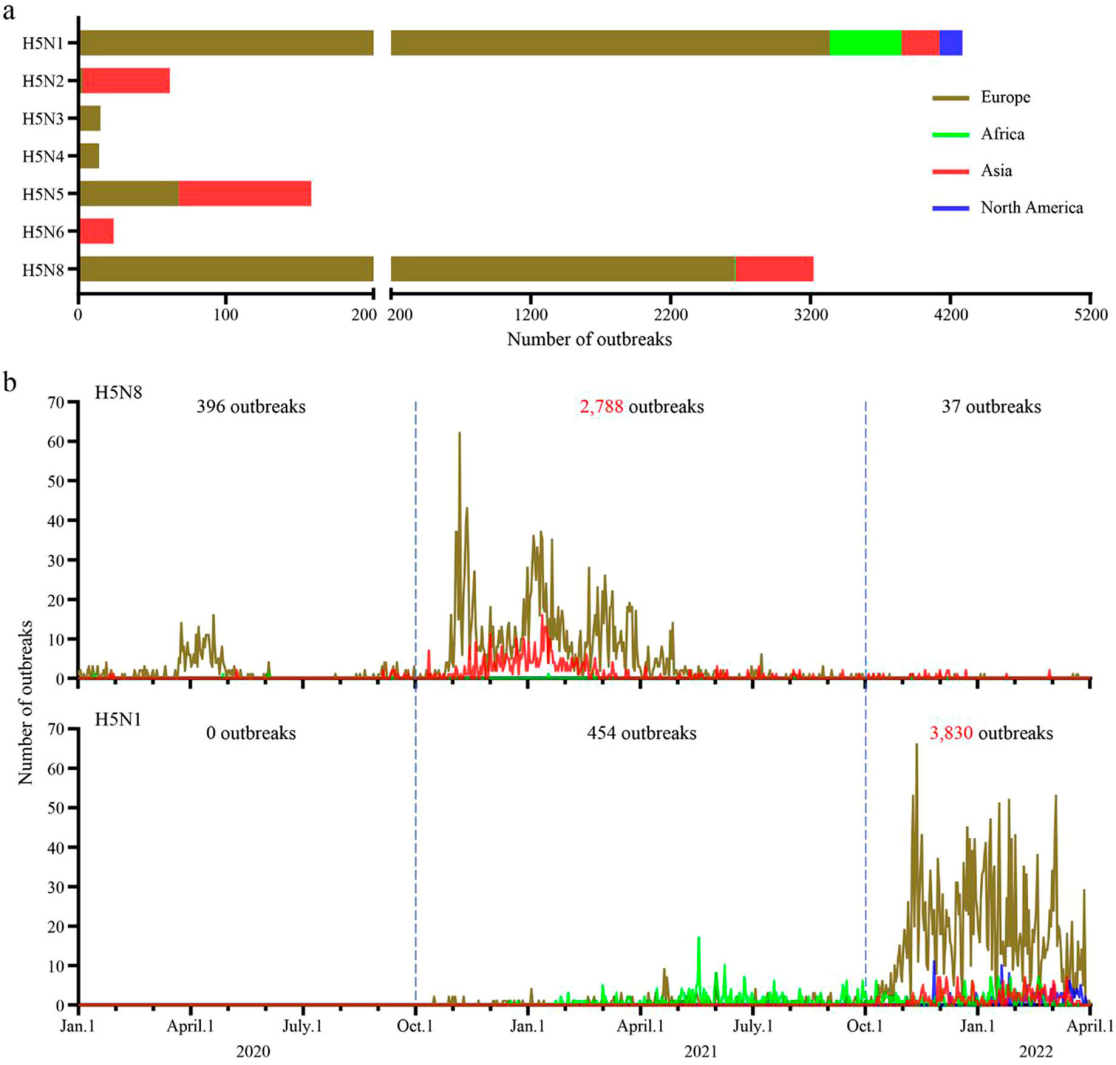
**Outbreaks and epidemiologic timeline of H5 avian influenza.** (a) The number of outbreaks caused by different subtypes of H5 viruses between January 1, 2020 and April 1, 2022. (b) The epidemiologic timeline of H5N1 and H5N8 viruses from January 1, 2020 to April 1, 2022. The epidemiology raw data for H5 highly pathogenic avian influenza over the indicated time period were downloaded from EMPRES-i + (https://empres-i.apps.fao.org/epidemiology).

Based on the epidemiologic data reported in EMPRES-i +, 7,778 outbreaks caused by different H5 highly pathogenic avian influenza viruses bearing the clade 2.3.4.4 HA gene occurred between January 2020 and March 2022: 4,284 outbreaks were caused by H5N1 viruses, 62 outbreaks were caused by H5N2 viruses, 15 outbreaks were caused by H5N3 viruses, 14 outbreaks were caused by H5N4 viruses, 158 outbreaks were caused by H5N5 viruses, 24 outbreaks were caused by H5N6 viruses, and 3,221 outbreaks were caused by H5N8 viruses (Figure 1(a)). The outbreaks caused by H5N2 and H5N6 viruses mainly occurred in Asia, the outbreaks caused by H5N3 and H5N4 viruses were only reported in Europe, and the H5N5 viruses caused outbreaks in both Europe and Asia. The outbreaks caused by H5N8 viruses were reported in countries in Europe, Africa, and Asia, and the outbreaks caused by H5N1 viruses were reported in countries in Europe, Africa, Asia, and North America (Figure 1(a)). Of note, 2,788 of the 3,221 outbreaks caused by H5N8 avian influenza viruses occurred between October 2020 and September 2021, and 3,830 of the 4,284 outbreaks caused by H5N1 viruses occurred after October 2021 (Figure 1(b)), indicating that the major strains causing global avian influenza outbreaks have shifted since October 2021 from the H5N8 to the H5N1 subtype.

### H5N1 viruses isolated in China

During our active surveillance of avian influenza viruses, a total of 811 avian influenza viruses were isolated from 26,767 samples that we collected between September 2021 and March 2022. These viruses belonged to nine different HA subtypes, including H1 (12 strains), H2 (2 strains), H3 (157 strains), H4 (24 strains), H5 (99 strains), H6 (214 strains), H9 (284 strains), H10 (14 strains), and H11 (5 strains). Thirteen of the 99 H5 viruses were confirmed as H5N1 subtype. Sequence analysis indicated that all of the H5N1 viruses bear the HA gene of the clade 2.3.4.4b. One virus was isolated from the lung tissue of a dead wild duck, and 12 viruses were isolated from chicken, duck, goose, and pigeon swabs collected in live poultry markets (Table 1). These viruses were isolated from samples collected from Anhui, Guangdong, Guizhou, Hebei, Hubei, Hunan, and Jiangxi provinces.

**Table 1.** H5N1 viruses bearing the clade 2.3.4.4b HA gene isolated in this study.

Virus			Sample information		
Full name	Abbreviation	Genotype	Sample type	Collected date	Location
A/wild duck/Hebei/SD012/2021(H5N1)	WD/HeB/SD012/2021	G1	Tissue	November 25, 2021	Wetland
A/duck/Hubei/S4465/2021(H5N1)	DK/HuB/S4465/2021	G9	Swab	November 27, 2021	Poultry market
A/chicken/Jiangxi/S40653/2021(H5N1)	CK/JX/S40653/2021	G9	Swab	December 7, 2021	Poultry market
A/duck/Jiangxi/S40833/2021(H5N1)	DK/JX/S40833/2021	G9	Swab	December 8, 2021	Poultry market
A/pigeon/Jiangxi/S40784/2021(H5N1)	PG/JX/S40784/2021	G9	Swab	December 8, 2021	Poultry market
A/duck/Guangdong/S4518/2021(H5N1)	DK/GD/S4518/2021	G7	Swab	December 8, 2021	Poultry market
A/duck/Guangdong/S4525/2021(H5N1)	DK/GD/S4525/2021	G7	Swab	December 8, 2021	Poultry market
A/goose/Hunan/SE284/2022(H5N1)	GS/HuN/SE284/2022	G10	Swab	January 5, 2022	Poultry market
A/duck/Hubei/SE128/2022(H5N1)	DK/HuB/SE128/2022	G9	Swab	January 10, 2022	Poultry market
A/duck/Hubei/SE220/2022(H5N1)	DK/HuB/SE220/2022	G7	Swab	January 10, 2022	Poultry market
A/duck/Guizhou/S1321/2022(H5N1)	DK/GZ/S1321/2022	G7	Swab	February 22, 2022	Poultry market
A/goose/Guizhou/S1541/2022(H5N1)	GS/GZ/S1541/2022	G7	Swab	February 22, 2022	Poultry market
A/chicken/Anhui/S1740/2022(H5N1)	CK/AH/S1740/2022	G9	Swab	March 3, 2022	Poultry market

### Phylogenic analysis of H5N1 viruses globally circulating since October 2020

To trace the origin of the H5N1 viruses isolated in China and understand their genetic relationship with those detected in other countries, we fully sequenced the whole genome of the 13 H5N1 viruses (the sequence data have been deposited in the GISAID EpiFlu Database under accession numbers EPI2029811–EPI2029914) and downloaded the genomic sequences of 220 H5N1 representative strains that were reported by others for phylogenetic and genotypic analyses.

**Figure 2.**
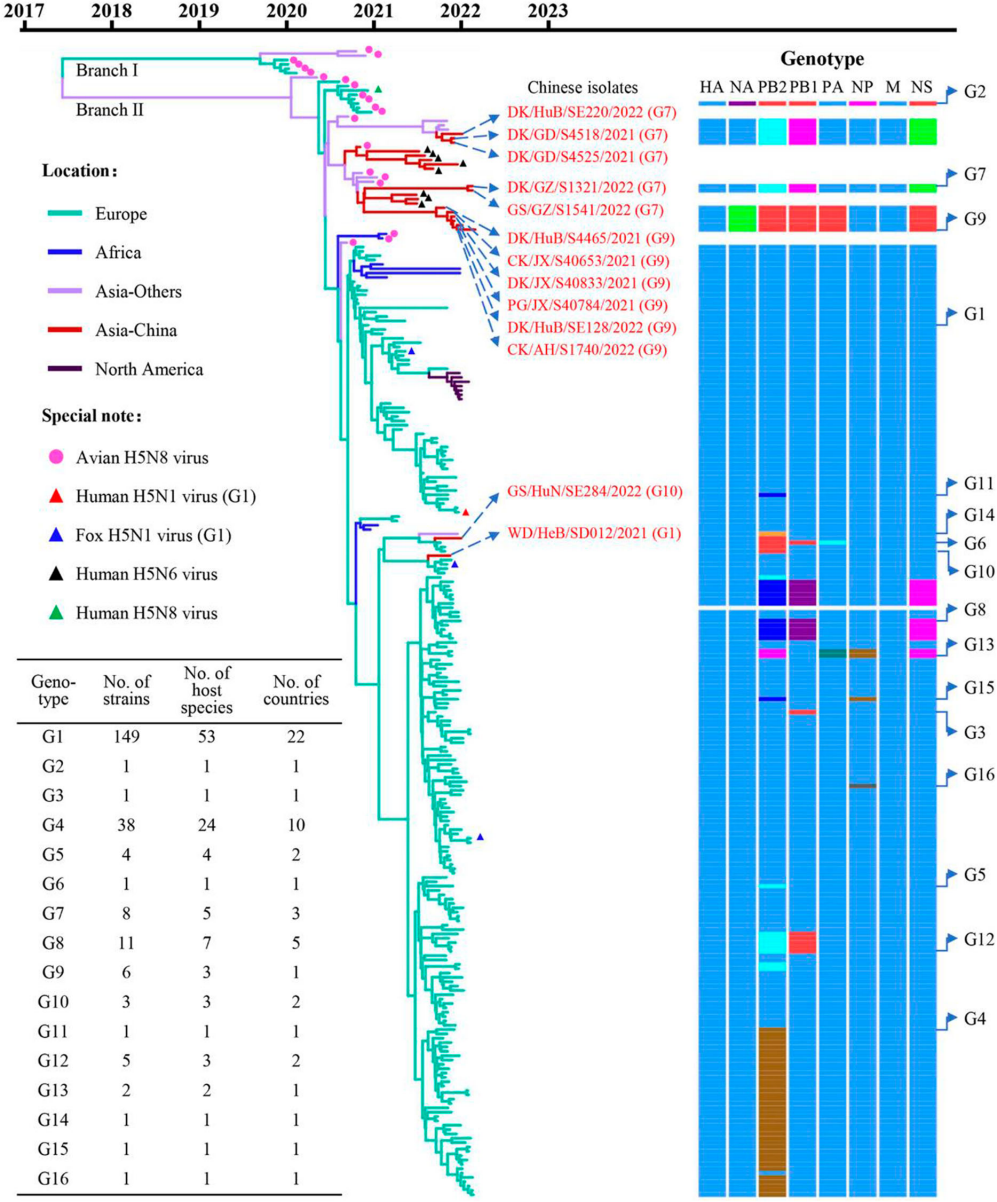
**Phylogenetic relationship of the globally circulating H5 viruses.** Bayesian time-resolved phylogenetic tree of the HA gene of 263 H5 viruses, including the 13 novel H5N1 viruses sequenced in this study and 250 H5 representative viruses downloaded from the GISAID EpiFlu Database (220 H5N1 viruses, eight H5N6 viruses, and 22 H5N8 viruses). The phylogenetic tree of the HA gene with more complete information is shown in Supporting Figure S1. The full name and location in the tree of the 13 H5N1 viruses detected in China are indicated in the Figure. The genotypes of the 233 H5N1 viruses were determined based on the diversity of their NA and internal genes, which are shown in Supporting Figure S2, and the closest gene donor of the NA and internal genes of these viruses are indicated in Figure 3. The eight bars represent the eight gene segments, and the colour of the bar indicates the closest donor strain of the gene segment. Detailed information about the host species in which the virus in each genotype was detected is provided in Supporting Table S1; detailed information about the country where the virus in each genotype was detected is provided in Supporting Table S2.

The clade 2.3.4.4b HA genes of H5 viruses have evolved into two branches [10], and we found that the HA genes of the 233 H5N1 viruses share 97.4%–100% identity at the nucleotide level and are all located in branch II of the Bayesian time-resolved phylogenetic tree (Figure 2). The HA genes of the 11 H5N1 viruses detected in this study clustered with the HA genes of H5N1, H5N6, and H5N8 viruses previously detected in Asia, and the HA genes of the other two viruses detected in this study clustered with the HA genes of H5N1 viruses detected in Europe (Figure 2, Figure S1). The NA genes of the 233 H5N1 viruses shared 88.8%–100% identity at the nucleotide level and formed three groups in the phylogenetic tree (Figure S2). The six internal genes of the 233 H5N1 viruses, that is, the basic polymerase 2 (PB2), basic polymerase 1 (PB1), acidic polymerase (PA), nucleoprotein (NP), matrix (M), and nonstructural protein (NS) genes, shared 89.4%–100%, 92.2%–100%, 94.0%–100%, 92.1%–100%, 98.1%–100%, and 70.6%–100% identity, respectively, at the nucleotide level and formed between one and seven groups in their phylogenetic trees (Figure S2). These results indicate that the NA and internal genes of the H5N1 viruses show clear diversity, and based on their genomic differences, the 233 H5N1 viruses detected in Europe, Africa, Asia, and North America formed 16 different genotypes (G1 to G16) (Figure 2). Of note, 149 of the 233 strains belong to G1, and have been detected in 53 different host species in 22 countries (Figure 2, Table S1, Table S2).

### Emergence and spatiotemporal spread of H5N1 viruses of each genotype

**Figure 3.**
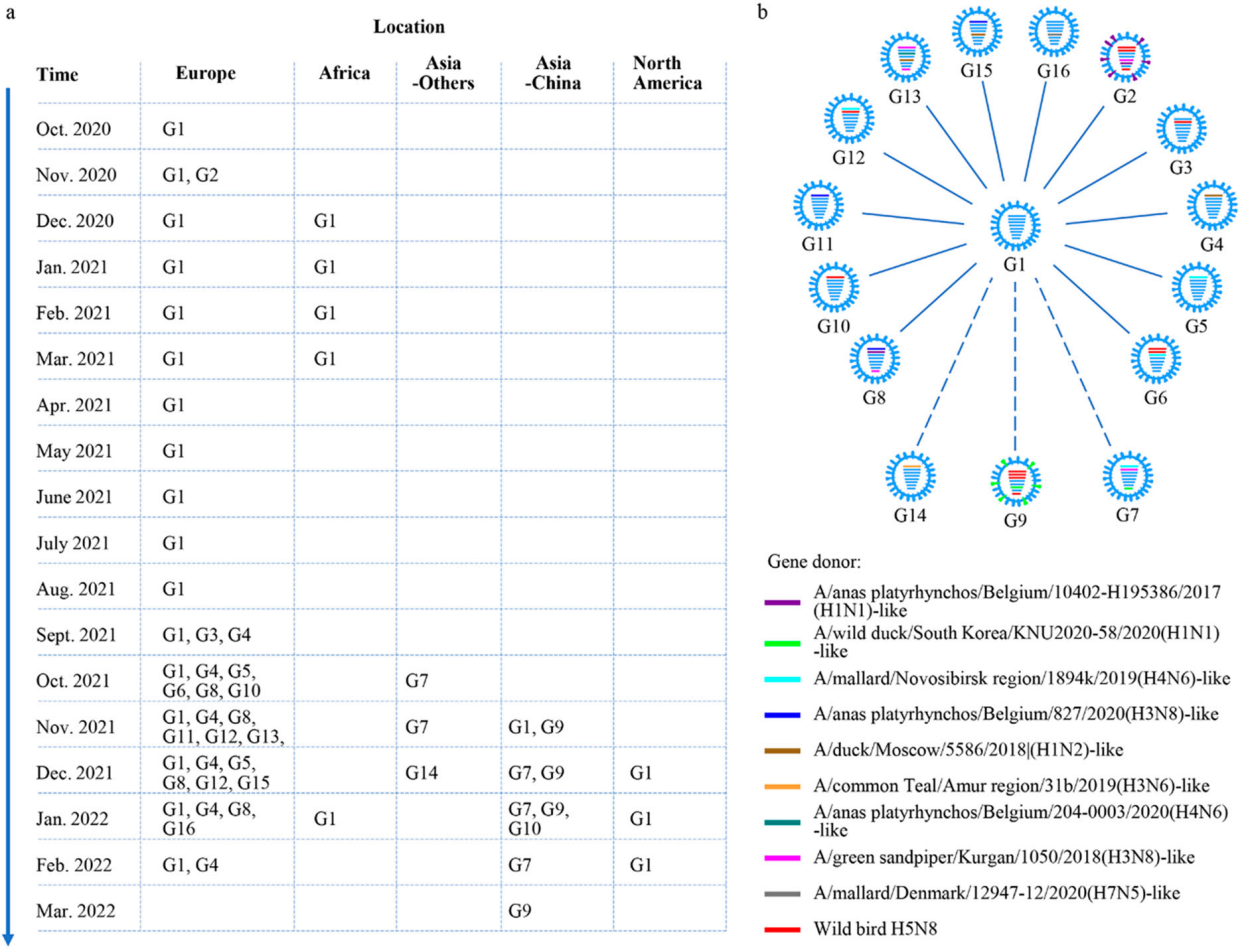
**The spatiotemporal spread of H5N1 viruses.** (a). Time and location where each genotype of H5N1 virus was detected. The countries in each continent where the viruses were detected are shown in Table S2. (b) Genotypic relationship of the H5N1 viruses. The eight bars represent the eight gene segments (from top to bottom: PB2, PB1, PA, HA, NP, NA, M, and NS); the colour of the bar indicates the closest donor strain of the gene segment.

To further understand the emergence, gene constellation, and spatiotemporal spread of the viruses in each genotype, we analyzed the time and location that each virus genotype was detected. We found that the G1 virus firstly emerged in Europe, specifically in the Netherlands [11] (Table S2), in October 2020, and circulated in European countries until February 2022 (Figure 3(a), Table S2). The G1 virus was also detected between December 2020 and March 2021, and in January 2022 in Africa (Figure 3(a)), in November 2021 in China, and between December 2021 and February 2022 in North America (Figure 3(a)). The G2 virus was only detected in the Netherlands in November 2020 (Table S2). The G3 to G6, G8, G11 to G13, G15, and G16 viruses were detected only in countries in Europe between September 2021 and February 2022 (Figure 3(a), Table S2). The G7 viruses were detected in different Asian countries between October 2021 and February 2022. The G9 viruses were detected only in China between November 2021 and March 2022, the G10 viruses were detected in Russia in October 2021 and in China in January 2022, and the G14 virus was detected only in the Asian country of Bangladesh in December 2021 (Figure 3(a), Table S2).

Detail genetic analysis indicates that the G1 virus is a reassortant of five different viruses: an H5N8 virus provided the HA and M genes, an A/gadwall/Chany/893/2018(H3N8)-like virus provided the PB2 and NP genes, an A/duck/Mongolia/217/2018(H3N8)-like virus provided the PB1 gene, an A/anas platyrhynchos/Belgium/10402-H195386/2017(H1N1)-like virus provided the PA and NS genes, and an A/anas platyrhynchos/Belgium/9594H191810/2016 (H1N1)-like virus provided the NA gene (Figure S3). To diagram the genetic relationship between the G1 genotype and the other genotypes, we used blue bars to show the gene segments that are similar to those of the G1 virus, and bars in other colours to show the gene segments that are different from those of the G1 virus in Figure 3(b). We found that the G2 virus has three genes (PA, HA, and M) that are similar to those of the G1 virus, whereas the other five genes are different. G3 virus has seven genes that are similar to those of the G1 virus, with only its PB1 differing from that of the G1 virus. G4, G5, G10, G11, and G14 viruses have seven genes that are similar to those of the G1 virus, with only their PB2 gene differing from those of the G1 virus. The G6, G7, and G8 viruses have five genes similar to those of the G1 virus, and three genes (G6: PB2, PB1, and PA; G7 and G8: PB2, PB1, and NS) that are different from those of G1 virus. The G9 virus has three genes (HA, NP, and M) similar to those of the G1 virus, while its other five genes differ. The G12 and G15 viruses have six genes that are similar to those of the G1 virus, and two genes (G12: PB2 and PB1; G15: PB2 and NP) that differ. The G13 virus has four genes (PB1, HA, NA, and M) similar to those of the G1 virus, and its PB2, PA, NP, and NS genes are different from those of the G1 virus. The G16 virus has seven genes that are similar to those of the G1 virus, with only its NP differing from that of the G1 virus (Figure 3(b)). Of note, the genes that are different from those of the G1 viruses were all derived from H5N8 or other low pathogenic viruses found in wild birds, indicating that reassortments have actively occurred in wild birds.

### Molecular analysis of the H5N1 viruses

All of these novel H5N1 viruses contain the polybasic amino acid motif of -RRKR/G- in their HA cleavage site, suggesting that all of these strains are highly pathogenic or potentially highly pathogenic to chickens [32,33]. A series of amino acid residues that have been reported to increase the virulence of avian influenza viruses in mammals were detected in all of these strains, including 225G in HA, 3V and 622G in PB1, 383D in PA, 437T in NP, 30D, 43M, and 215A in M1, and 106M in NS1 (Table S3). Several other amino acid residues responsible for increased virulence in mice were also detected in some of the strains, specifically 292V in PB2 in 11 strains, 627K in PB2 in one strain, 389R in PB2 in 219 strains, 482R in PB2 in seven strains, 598T in PB2 in 232 strains, 37A in PA in 232 strains, 63I in PA in three strains, 286A in NP in 186 strains, 319K in NP in seven strains, and 42S in NS1 in 220 strains (Table S3). These analyses suggest that the H5N1 viruses may be able to infect and cause disease in mammals.

### Replication and virulence of H5N1 viruses in mice

**Figure 4.**
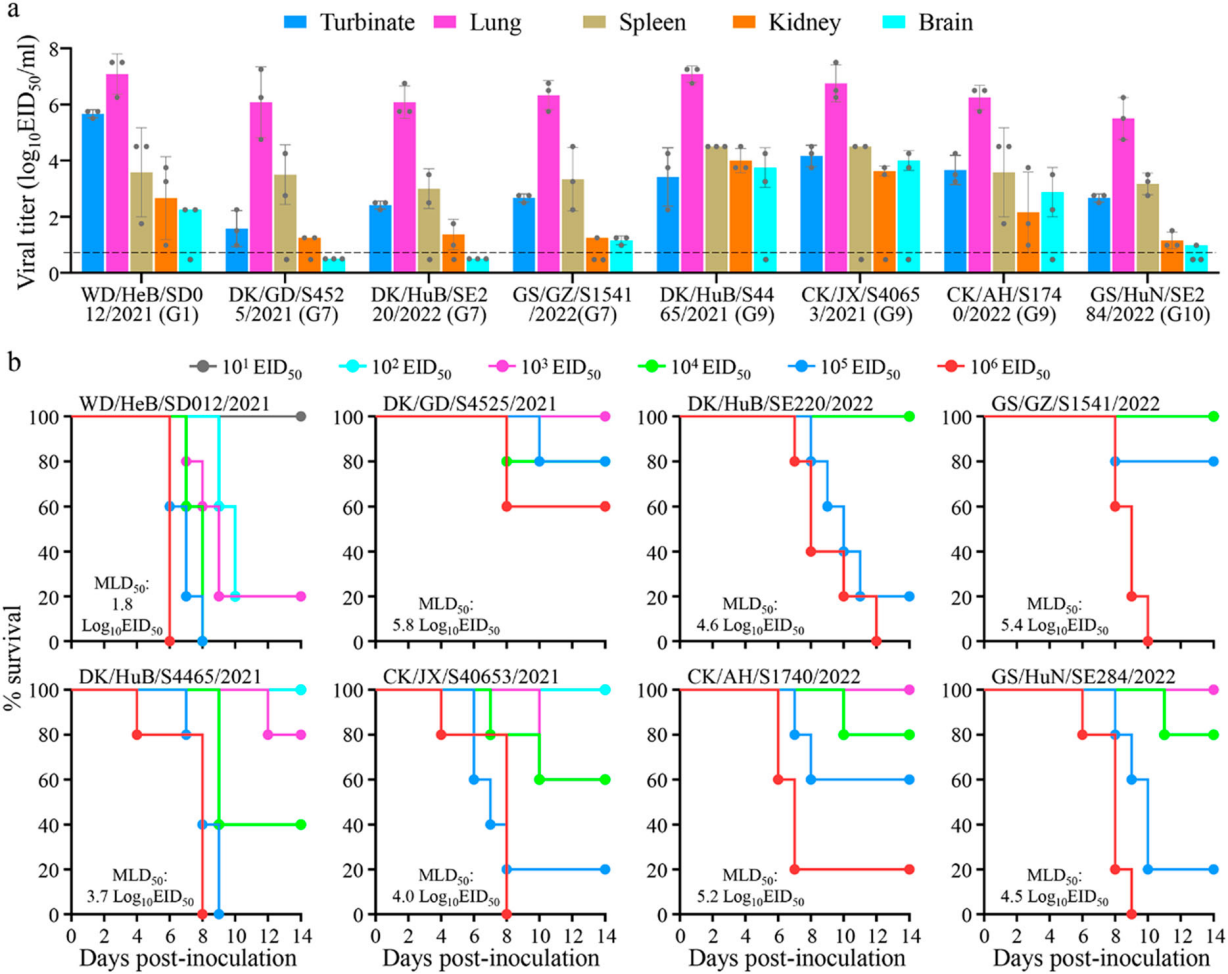
**Replication and virulence of H5N1 viruses in mice.** (a) Virus titers in organs of mice inoculated intranasally with 10^6^ EID_50_ of different H5N1 viruses. Three mice from each group were euthanized and their organs were collected on day 3 post-inoculation for virus titration in eggs. Data shown are means ± standard deviations. The dashed line indicates the lower limit of detection. (b) Death pattern and MLD_50_ values of the indicated viruses.

To evaluate the replication and virulence of the H5N1 viruses in mammals, we tested eight representative strains detected in China in mice, including one G1 virus, three G7 viruses, three G9 viruses, and one G10 virus. We found that six of these viruses––WD/HeB/SD012/2021, GS/GZ/S1541/2022, DK/HuB/S4465/2021, CK/JX/S40653/2021, CK/AH/S1740/2022, and GS/HuN/SE284/2022––replicated systemically and were detected in all five tested organs of mice (Figure 4(a)); their 50% mouse lethal doses (MLD_50_) were 1.8 log_10_ 50% egg infectious doses (EID_50_), 5.4 log_10_ EID_50_, 3.7 log_10_ EID_50_, 4.0 log_10_ EID_50_, 5.2 log_10_ EID_50_, and 4.5 log_10_ EID_50_, respectively (Figure 4(b)). DK/GD/S4525/2021 and DK/HuB/SE220/2022 were detected in the nasal turbinate, lungs, spleen, and kidneys, but not in the brain of any mice (Figure 4(a)); their MLD_50_ values were 5.8 log_10_ EID_50_ and 4.6 log_10_ EID_50_, respectively (Figure 4(b)). These results indicate that H5N1 viruses detected in China have distinct pathotypes in mice, and that the G1 virus WD/HeB/SD012/2021 is the most virulent of the viruses tested in mice.

### Cross-reactivity of H5N1 viruses with antisera induced by different vaccine seed viruses

**Figure 5.**
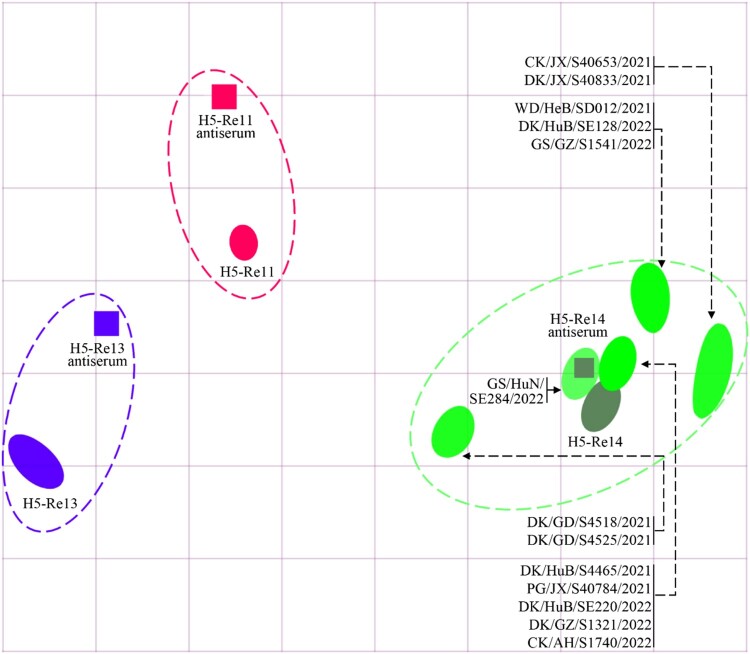
**Antigenic cartography of H5N1 viruses.** The antigenic map was generated by using the HI assay data shown in Table S4. Each unit in the coordinate represents a 2-fold difference in HI titer. The squares represent the antisera generated from the indicated viruses. The different coloured ovals show the viruses used for antisera generation and the test viruses.

Vaccination is the major strategy for the control of highly pathogenic avian influenza in China, and we have developed different vaccine strains to prevent infection with viruses in different clades or subclades. The H5-Re11 vaccine seed virus was developed to target H5 viruses bearing the clade 2.3.4.4h HA [31,34], and was used for vaccine production between December 2018 and December 2021. H5-Re13 and H5-Re14 are recently updated vaccine seed viruses that were developed to target the newly detected H5 viruses bearing the clade 2.3.4.4h HA and the clade 2.3.4.4b HA gene, respectively, and have been used for vaccine production since January 2022 [30,35]. We performed an HI assay to investigate the cross-reactivity of antisera induced by these vaccine seed viruses against the 13 H5N1 viruses. We found that the HI antibody titers of H5-Re11, H5-Re13, and H5-Re14 antisera against the homologous viruses were 1024, 1024, and 512, respectively (Table S4). The HI titers of the H5-Re11 antiserum against the 13 H5N1 viruses were 16–32, which was 32- to 64-fold lower than the homologous titer; the HI titers of the H5-Re13 antiserum against the 13 H5N1 viruses were 16–256, which was 4- to 64-fold lower than the homologous titer; however, the HI titers of the H5-Re14 antiserum against the H5N1 viruses were 256–512, which was the same as the homologous titer or only 2-fold lower (Figure 5, Table S4). These results indicate that antisera induced by vaccine seed viruses bearing the clade 2.3.4.4h HA gene react poorly with the newly emerged H5N1 viruses and that antisera induced by the H5-Re14 vaccine seed virus cross-reacts well with the H5N1 viruses, suggesting that the currently used vaccine may provide adequate protection against the H5N1 viruses.

## Discussion

H5N1 viruses bearing the clade 2.3.4.4b HA gene have become the predominant strains causing global avian influenza outbreaks since October 2021. In this study, we performed phylogenic analyses of 220 H5N1 viruses that were reported in 27 countries together with 13 viruses we isolated in China, and found that the globally circulating H5N1 viruses have formed 16 different genotypes. The G1 viruses are the most widely circulating strains having been detected in wild birds or domestic poultry in 22 countries across Europe, Africa, Asia, and North America. The 13 H5N1 viruses isolated in China belong to the G1, G7, G9, and G10 genotypes, and viruses of all four of these genotypes replicated efficiently in multiple organs of mice, although their pathogenicity varied among strains. Moreover, the newly detected H5N1 viruses antigenically matched the H5-Re14 vaccine seed virus that is currently used in China to prevent infection with H5 viruses bearing clade 2.3.4.4b HA.

Reassortment is the major mechanism for influenza virus evolution. During their circulation in nature, H5N8 viruses bearing clade 2.3.4.4b HA reassorted with four different low pathogenic viruses and generated the G1 H5N1 viruses, and these G1 H5N1 viruses further reassorted with at least 10 different avian influenza viruses, thereby formed 15 more H5N1 virus genotypes. Of note, our analyses indicated that 14 of the 16 genotypes emerged between September 2021 and January 2022 (Figure 3(a), Table S2), which further demonstrates that the avian influenza viruses are more active in winter.

Gu et al. detected different genotypes of H5N6 viruses in China and found that the H5N6 viruses were reassortants that derived their HA genes from H5N8 viruses, NA genes from different duck H5N6 viruses, and certain internal genes from different low pathogenic viruses [15]. Most of the donor viruses of the NA and internal genes of the H5N6 viruses were previously detected in China, suggesting that the H5N6 reassortants were generated in ducks in China [15]. However, it seems that the four genotypes of the H5N1 viruses detected in China have not undergone any further reassortment in domestic birds in China. Among the four genotypes of H5N1 viruses in China, three of them (G1, G7, and G10) previously appeared in other countries; the G9 viruses have only been detected in China. Since the donor of the NA gene of the G9 viruses was previously detected in wild ducks in South Korea (Figure 3(b)), it is reasonable to speculate that the G9 virus was originally generated in wild birds in this region and then spread to domestic ducks in China, although there have been no reports of G9 virus detection in wild birds.

The H5 avian influenza viruses are becoming progressively more virulent in the mammalian mouse model. Some of the earliest H5N1 viruses could not replicate in mice [36], but more and more strains have now acquired this ability and some have become lethal in mice [4,10,15]. A series of amino acid substitutions in different proteins of avian influenza virus have been reported to increase its pathogenicity to mice [37–53], and many of these substitutions have been detected in the widely circulating H5N1 viruses, which explains why the H5N1 viruses we tested in this study were able to replicate in and kill mice. The PB2 E627K substitution was found to increase the pathogenicity of H5N1, H7N9, and H9N2 viruses in mice [42,54,55], and to promote the respiratory droplet transmission of H1N1, H5N1, H7N9, and H9N2 viruses in ferrets [54–57]. The widely spread G1 viruses have caused human infection in the United Kingdom and fox infection in Ireland, Estonia, and the Netherlands, and the virus isolated from a fox in Ireland had PB2 627K (GISAID accession # EPI1998135). These facts suggest that the H5N1 viruses could become more dangerous if they have the opportunity to replicate in mammals, and therefore, they should be carefully monitored and evaluated in mammals.

H5N1 viruses have caused severe damage to the global poultry industry, with more than 70 million domestic poultry having been destroyed in efforts to contain the disease since October 2020 [58]. Of note, about 30 million poultry have been destroyed in the United States alone [58]. After detection of H5N8 viruses bearing clade 2.3.4.4b HA in China [10], an updated H5/H7 trivalent vaccine was produced with the H5-Re13, H5-Re14, and H7-Re4 seed viruses and has been used in China since January 2022 [30,35]. The H5-Re14 seed virus was developed to protect against H5 viruses bearing the clade 2.3.4.4b HA gene, and a challenge study by Zeng et al. [30] showed that the novel H5/H7 trivalent vaccine can provide solid protection against challenge with H5N1, H5N6, and H5N8 viruses bearing clade 2.3.4.4b HA. Our antigenic analysis in this study demonstrated that H5-Re14 antigenically matches well with the H5N1 viruses of four different genotypes. Given the wide circulation of H5N1 viruses in wild birds, it is highly likely that these viruses will continue to harm the poultry industry and pose a threat to human public health. Therefore, we strongly recommend that high-risk countries vaccinate their poultry to protect them against highly pathogenic H5 avian influenza.

## Supplemental information

Supplemental information comprising three figures and four tables can be found with this article online at the journal’s website.

## Supplementary Material

Supplemental Material
