# Supplementary material for: Global dissemination of H5N1 influenza viruses bearing the clade 2.3.4.4b HA gene and biologic analysis of the ones detected in China
Source: Emerg Microbes Infect. 2022 Jun 28;11(1):1693–704. doi: 10.1080/22221751.2022.2088407 (PMC9246030; doi:10.1080/22221751.2022.2088407)
Supplement: Supplemental Material [file TEMI_A_2088407_SM9124.zip › Cui Table S1.docx]

**Table S1. Host species infected by the H5N1 viruses.**

| Genotype | No. of host species | Host |
| --- | --- | --- |
| G1 | 53 | American blue-winged teal, American wigeon, Anser albifrons, barnacle goose, bean goose, black-headed gull, buzzard, Caspian gull, chicken, common murre, crow, duck, human, Eurasian eagle-owl, Eurasian teal, Eurasian wigeon, European herring gull, fox, gadwall, goose, great black-backed gull, great skua, greater white-fronted goose, great-white pelican, grey heron, graylag goose, gull, herring gull, kestrel, magpie, mallard, Muscovy duck, mute swan, northern goshawk, northern pintail, northern shoveler, owl, pelican, pheasant, quail, rooster, sanderling, seagull, stork, swan, turkey, western jackdaw, western marsh harrier, white-tailed eagle, whooper swan, wild goose, withe-tiled eagle, wild duck |
| G2 | 1 | Eurasian curlew |
| G3 | 1 | turkey |
| G4 | 24 | Anser anser, barnacle goose, Branta leucopsis, brent goose, Caspian gull, chicken, common crane, duck, Eurasian curlew, European herring gull, gallus, goose, gray heron, great black-backed gull, heron, herring gull, mallard, mute swan, oystercatcher, peregrine falcon, stork, turkey, western jackdaw, white-fronted goose |
| G5 | 4 | chicken, goose, seagull, swan |
| G6 | 1 | chicken |
| G7 | 5 | chicken, mandarin duck, quail, duck, goose |
| G8 | 7 | chicken, Cygnus olor, duck, egret, goose, partridge, turkey |
| G9 | 3 | chicken, duck, pigeon |
| G10 | 3 | chicken, turkey, goose |
| G11 | 1 | buzzard |
| G12 | 3 | chicken, mute swan, turkey |
| G13 | 2 | great egret, grey heron |
| G14 | 1 | duck |
| G15 | 1 | goose |
| G16 | 1 | peregrine falcon |
