# Supplementary material for: Global dissemination of H5N1 influenza viruses bearing the clade 2.3.4.4b HA gene and biologic analysis of the ones detected in China
Source: Emerg Microbes Infect. 2022 Jun 28;11(1):1693–704. doi: 10.1080/22221751.2022.2088407 (PMC9246030; doi:10.1080/22221751.2022.2088407)
Supplement: Supplemental Material [file TEMI_A_2088407_SM9124.zip › Cui Table S2.docx]

**Table S2. Spatiotemporal spread of the H5N1 viruses in each genotype.**

| **Continent** | **Country** | **2020** | | | **2021** | | | | | | | | | | | | **2022** | | |
| --- | --- | --- | --- | --- | --- | --- | --- | --- | --- | --- | --- | --- | --- | --- | --- | --- | --- | --- | --- |
|  |  | **Oct.** | **Nov.** | **Dec.** | **Jan.** | **Feb.** | **Mar.** | **Apr.** | **May** | **June** | **July** | **Aug.** | **Sep.** | **Oct.** | **Nov.** | **Dec.** | **Jan.** | **Feb.** | **Mar.** |
| Europe | Netherlands | G1 | G1,  G2 | G1 | G1 | G1 | G1 | G1 | G1 |  |  |  |  | G1,  G4 | G1,  G4,  G11 | G1,  G4 | G1,  G4 | G1,  G4 |  |
|  | Italy |  | G1 |  |  |  |  |  |  |  |  |  |  | G1,  G8 | G1,  G8,  G12 | G1,  G4,  G8,  G12 |  |  |  |
|  | UK |  |  | G1 |  | G1 |  |  |  |  |  | G1 |  | G1 | G1 | G1 |  |  |  |
|  | Denmark |  |  |  |  |  | G1 |  |  |  |  |  |  |  |  |  |  |  |  |
|  | Finland |  |  |  |  |  |  | G1 |  | G1 | G1 |  |  |  |  |  |  |  |  |
|  | Hungary |  |  |  |  |  |  | G1 |  |  |  |  |  |  |  |  |  |  |  |
|  | Poland |  |  |  |  |  |  | G1 |  |  |  |  |  |  | G1 |  |  |  |  |
|  | Estonia |  |  |  |  |  |  |  | G1 | G1 |  |  |  | G1 | G1 |  |  |  |  |
|  | Sweden |  |  |  |  |  |  |  | G1 |  |  |  | G1 | G1 | G1,  G4 | G1 |  |  |  |
|  | Czech  Republic |  |  |  |  |  |  |  |  |  |  |  | G1 |  | G1,  G12,  G13 | G1,  G12 | G1 |  |  |
|  | Belgium |  |  |  |  |  |  |  |  |  |  |  |  |  | G1,  G4 | G4 | G4 |  |  |
|  | Bulgaria |  |  |  |  |  |  |  |  |  |  |  |  |  | G8 | G8 |  |  |  |
|  | Croatia |  |  |  |  |  |  |  |  |  |  |  |  |  | G1,  G4 |  |  |  |  |
|  | France |  |  |  |  |  |  |  |  |  |  |  |  |  | G1,  G4 | G1,  G8,  G15 |  |  |  |
|  | Ireland |  |  |  |  |  |  |  |  |  |  |  |  |  | G1,  G4 | G1 | G1,  G16 | G1 |  |
|  | Romania |  |  |  |  |  |  |  |  |  |  |  |  |  | G4,  G8 |  |  |  |  |
|  | Slovenia |  |  |  |  |  |  |  |  |  |  |  |  |  |  | G1,  G5 | G1 |  |  |
|  | Spain |  |  |  |  |  |  |  |  |  |  |  |  |  |  | G1 | G1,  G4 | G4 |  |
|  | Moldova |  |  |  |  |  |  |  |  |  |  |  |  |  |  |  | G8 |  |  |
|  | Russia |  |  |  |  |  |  |  |  |  |  |  | G1,  G3,  G4 | G1,  G5,  G6,  G10 | G1 | G5 |  |  |  |
| Africa | Senegal |  |  | G1 | G1 |  |  |  |  |  |  |  |  |  |  |  |  |  |  |
|  | Nigeria |  |  |  |  | G1 | G1 |  |  |  |  |  |  |  |  |  |  |  |  |
|  | Niger |  |  |  |  |  |  |  |  |  |  |  |  |  |  |  | G1 |  |  |
| Asia | Korea |  |  |  |  |  |  |  |  |  |  |  |  | G7 | G7 |  |  |  |  |
|  | Japan |  |  |  |  |  |  |  |  |  |  |  |  |  | G7 |  |  |  |  |
|  | China |  |  |  |  |  |  |  |  |  |  |  |  |  | G1,  G9 | G7,  G9 | G7,  G9,  G10 | G7 | G9 |
|  | Bangladesh |  |  |  |  |  |  |  |  |  |  |  |  |  |  | G14 |  |  |  |
| North  America | USA |  |  |  |  |  |  |  |  |  |  |  |  |  |  | G1 | G1 | G1 |  |
