# Supplementary material for: Global dissemination of H5N1 influenza viruses bearing the clade 2.3.4.4b HA gene and biologic analysis of the ones detected in China
Source: Emerg Microbes Infect. 2022 Jun 28;11(1):1693–704. doi: 10.1080/22221751.2022.2088407 (PMC9246030; doi:10.1080/22221751.2022.2088407)
Supplement: Supplemental Material [file TEMI_A_2088407_SM9124.zip › Cui Table S3.docx]

**Table S3. Mutations detected in the H5N1 viruses that are known to contribute to increased replication and virulence in mammals.**

| Genotype  (No. of strains) | Amino acids that increase the replication and virulence of avian influenza viruses in mammals | | | | | | | | | | | | | | | | | | | |
| --- | --- | --- | --- | --- | --- | --- | --- | --- | --- | --- | --- | --- | --- | --- | --- | --- | --- | --- | --- | --- |
|  | HA |  | PB2 | | | |  | PB1 |  | PA | |  | NP | | |  | M1 |  | NS1 | |
|  | 225G^a^ |  | 292V | 389R/598T | 482R | 627K |  | 3V/622G |  | 37A/383D | 63I |  | 286A | 319K | 437T |  | 30D/43M/215A |  | 42S | 106M |
| G1 (149) | 149 |  | /^b^ | 135/148 | 7 | 1 |  | 146^c^/149 |  | 149/149 | / |  | 103 | 7 | 149 |  | 149/149/149 |  | 149 | 149 |
| G2 (1) | 1 |  | 1 | 1/1 | / | / |  | 1/1 |  | 1/1 | / |  | / | / | 1 |  | 1/1/1 |  | 1 | 1 |
| G3 (1) | 1 |  | / | 1/1 | / | / |  | 1/1 |  | 1/1 | / |  | 1 | / | 1 |  | 1/1/1 |  | 1 | 1 |
| G4 (38) | 38 |  | / | 38/38 | / | / |  | 38/38 |  | 38/38 | / |  | 38 | / | 38 |  | 38/38/38 |  | 38 | 38 |
| G5 (4) | 4 |  | / | 4/4 | / | / |  | 4/4 |  | 4/4 | / |  | 4 | / | 4 |  | 4/4/4 |  | 4 | 4 |
| G6 (1) | 1 |  | 1 | 1/1 | / | / |  | 1/1 |  | 1/1 | / |  | 1 | / | 1 |  | 1/1/1 |  | 1 | 1 |
| G7 (8) | 8 |  | / | 8/8 | / | / |  | 8/8 |  | 8/8 | / |  | 8 | / | 8 |  | 8/8/8 |  | 8 | 8 |
| G8 (11) | 11 |  | / | 11/11 | / | / |  | 11/11 |  | 11/11 | / |  | 11 | / | 11 |  | 11/11/11 |  | / | 11 |
| G9 (6) | 6 |  | 6 | 6/6 | / | / |  | 6/6 |  | 5/6 | / |  | 6 | / | 6 |  | 6/6/6 |  | 6 | 6 |
| G10 (3) | 3 |  | 3 | 3/3 | / | / |  | 3/3 |  | 3/3 | 3 |  | 3 | / | 3 |  | 3/3/3 |  | 3 | 3 |
| G11 (1) | 1 |  | / | 1/1 | / | / |  | 1/1 |  | 1/1 | / |  | 1 | / | 1 |  | 1/1/1 |  | 1 | 1 |
| G12 (5) | 5 |  | / | 5/5 | / | / |  | 5/5 |  | 5/5 | / |  | 5 | / | 5 |  | 5/5/5 |  | 5 | 5 |
| G13 (2) | 2 |  | / | 2/2 | / | / |  | 2/2 |  | 2/2 | / |  | 2 | / | 2 |  | 2/2/2 |  | / | 2 |
| G14 (1) | 1 |  | / | 1/1 | / | / |  | 1/1 |  | 1/1 | / |  | 1 | / | 1 |  | 1/1/1 |  | 1 | 1 |
| G15 (1) | 1 |  | / | 1/1 | / | / |  | 1/1 |  | 1/1 | / |  | 1 | / | 1 |  | 1/1/1 |  | 1 | 1 |
| G16 (1) | 1 |  | / | 1/1 | / | / |  | 1/1 |  | 1/1 | / |  | 1 | / | 1 |  | 1/1/1 |  | 1 | 1 |

a, H3 numbering. b, No such mutant. c, N-terminal sequences of the PB1 gene of three viruses in genotype G1 are not available.
