# Supplementary material for: Global dissemination of H5N1 influenza viruses bearing the clade 2.3.4.4b HA gene and biologic analysis of the ones detected in China
Source: Emerg Microbes Infect. 2022 Jun 28;11(1):1693–704. doi: 10.1080/22221751.2022.2088407 (PMC9246030; doi:10.1080/22221751.2022.2088407)
Supplement: Supplemental Material [file TEMI_A_2088407_SM9124.zip › Cui Table S4.docx]

**Table S4. Cross-reactive HI antibody titers of the H5N1 viruses with antisera induced by different vaccine seed viruses.**

| Virus | Clade | HI antibody titer of antiserum induced by different antigens ^a^ | | |
| --- | --- | --- | --- | --- |
|  |  | H5-Re11 | H5-Re13 | H5-Re14 |
| H5-Re11 | 2.3.4.4h | **1024** | 2048 | 128 |
| H5-Re13 | 2.3.4.4h | 32 | **1024** | 8 |
| H5-Re14 | 2.3.4.4b | 16 | 64 | **512** |
| WD/HeB/SD012/2021 (G1) | 2.3.4.4b | 32 | 32 | 256 |
| DK/GD/S4518/2021 (G7) | 2.3.4.4b | 32 | 256 | 256 |
| DK/GD/S4525/2021 (G7) | 2.3.4.4b | 32 | 256 | 256 |
| DK/HuB/SE220/2022 (G7) | 2.3.4.4b | 32 | 32 | 512 |
| DK/GZ/S1321/2022 (G7) | 2.3.4.4b | 32 | 32 | 512 |
| GS/GZ/S1541/2022 (G7) | 2.3.4.4b | 32 | 32 | 256 |
| DK/HuB/S4465/2021 (G9) | 2.3.4.4b | 32 | 32 | 512 |
| CK/JX/S40653/2021 (G9) | 2.3.4.4b | 16 | 16 | 256 |
| DK/JX/S40833/2021 (G9) | 2.3.4.4b | 16 | 16 | 256 |
| PG/JX/S40784/2021 (G9) | 2.3.4.4b | 32 | 32 | 512 |
| DK/HuB/SE128/2022 (G9) | 2.3.4.4b | 32 | 32 | 256 |
| CK/AH/S1740/2022 (G9) | 2.3.4.4b | 32 | 32 | 512 |
| GS/HuN/SE284/2022 (G10) | 2.3.4.4b | 32 | 64 | 512 |

a. Antisera were generated by vaccinating specific-pathogen-free chickens with the indicated oil-emulsified inactivated viruses; the homologous titers are shown in bold.
