# Supplementary figures and images for: Global dissemination of H5N1 influenza viruses bearing the clade 2.3.4.4b HA gene and biologic analysis of the ones detected in China
Source: Emerg Microbes Infect. 2022 Jun 28;11(1):1693–704. doi: 10.1080/22221751.2022.2088407 (PMC9246030; doi:10.1080/22221751.2022.2088407)

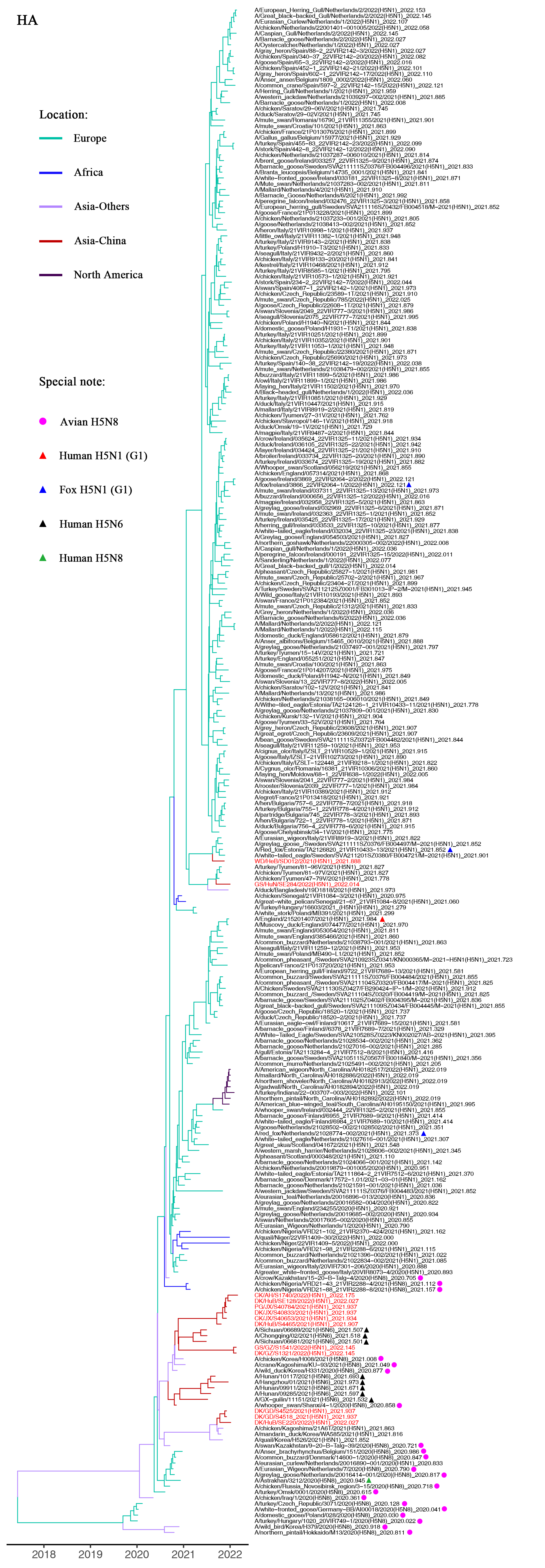

Supplement: Supplemental Material [file TEMI_A_2088407_SM9124.zip › Cui Fig S1.tif]

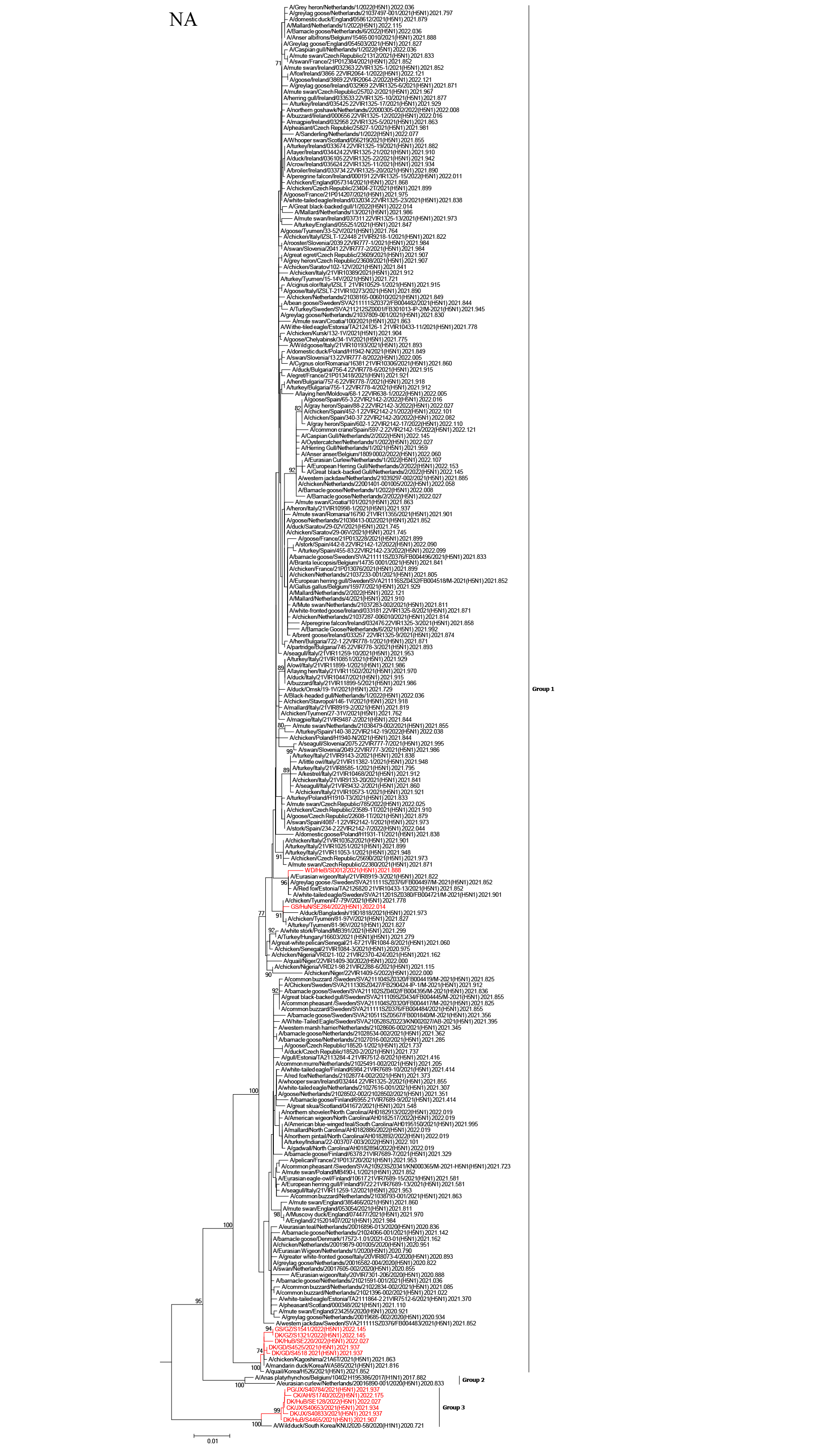

Supplement: Supplemental Material [file TEMI_A_2088407_SM9124.zip › Cui Fig S2a.tif]

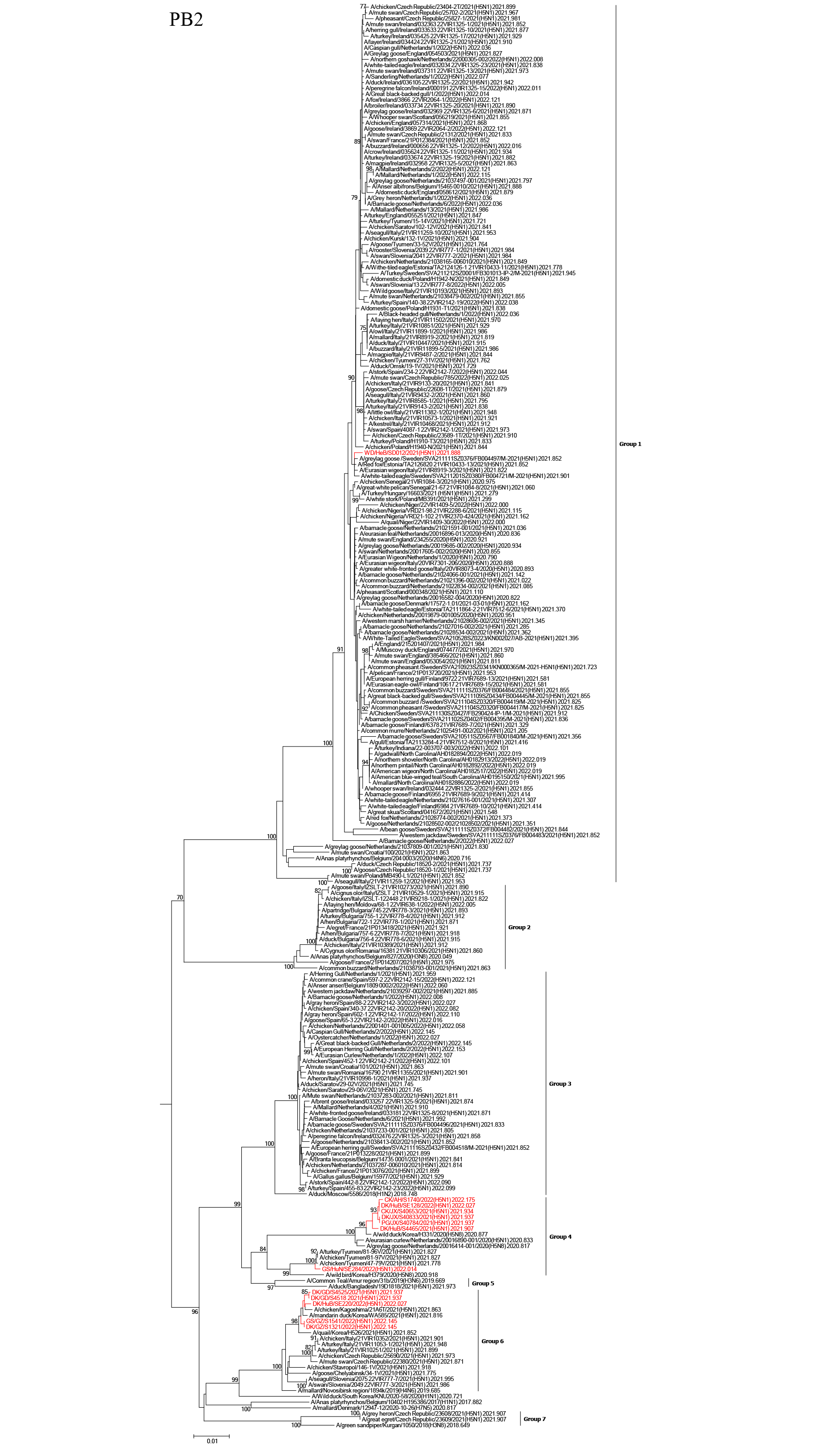

Supplement: Supplemental Material [file TEMI_A_2088407_SM9124.zip › Cui Fig S2b.tif]

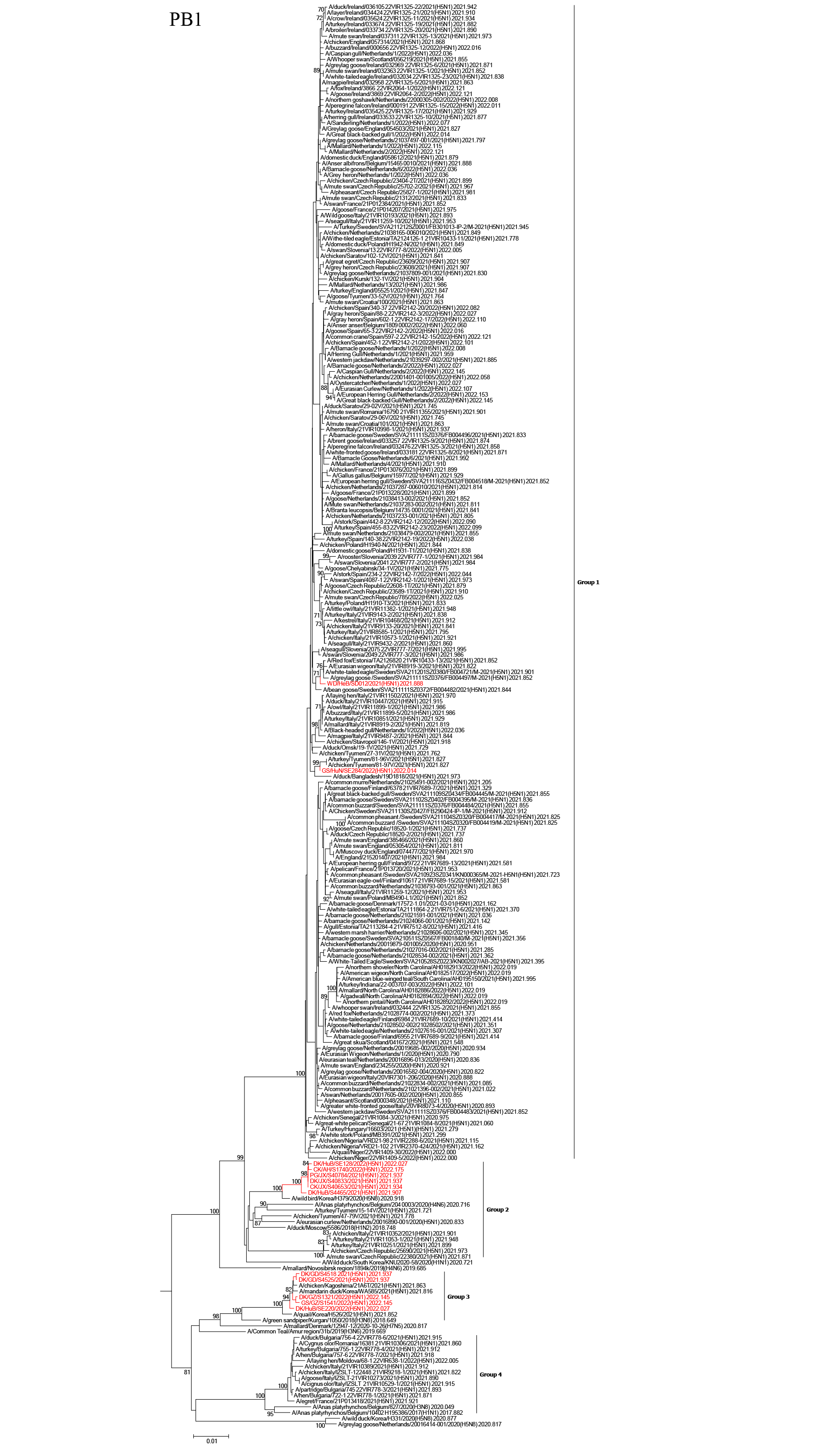

Supplement: Supplemental Material [file TEMI_A_2088407_SM9124.zip › Cui Fig S2c.tif]

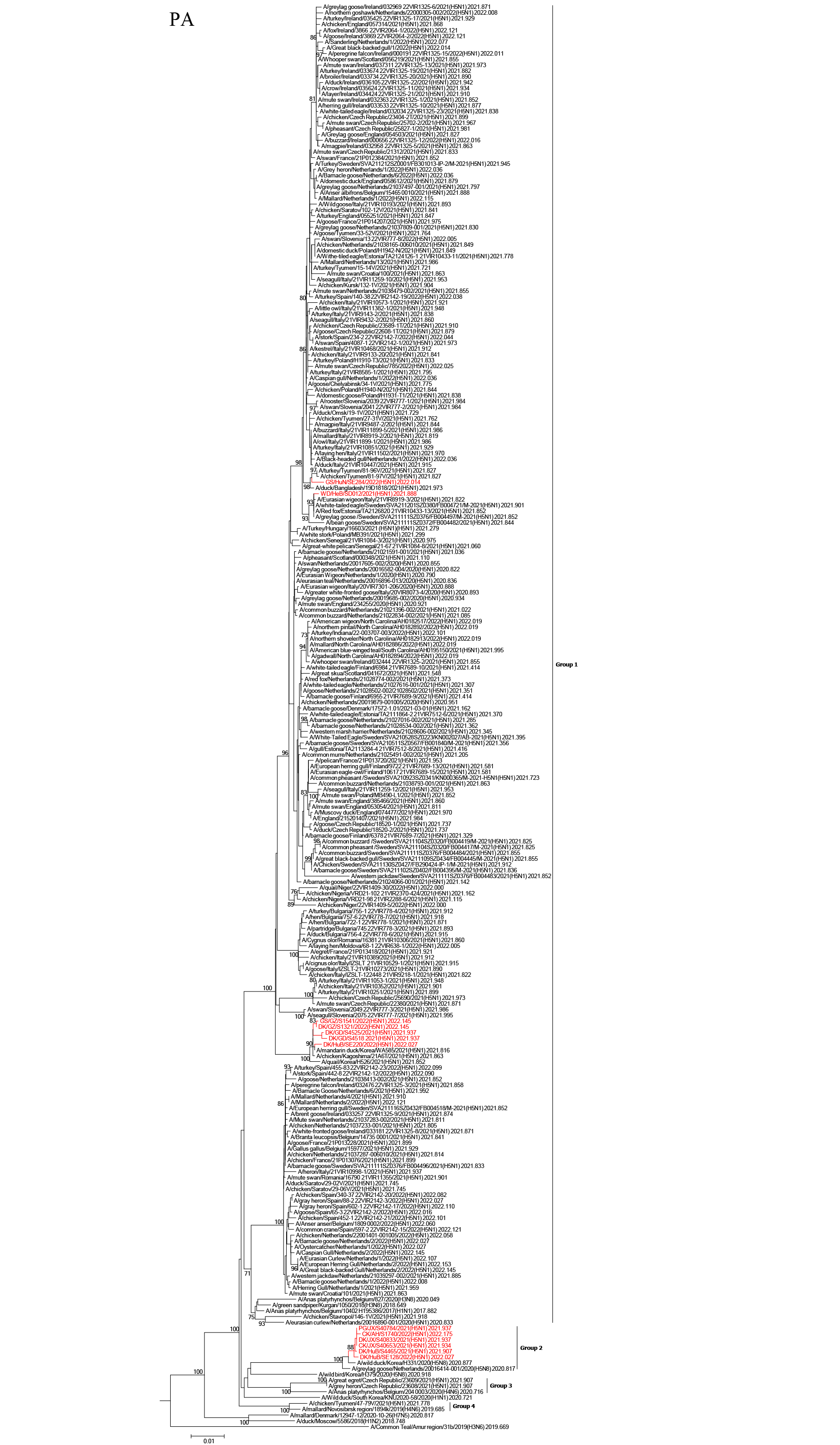

Supplement: Supplemental Material [file TEMI_A_2088407_SM9124.zip › Cui Fig S2d.tif]

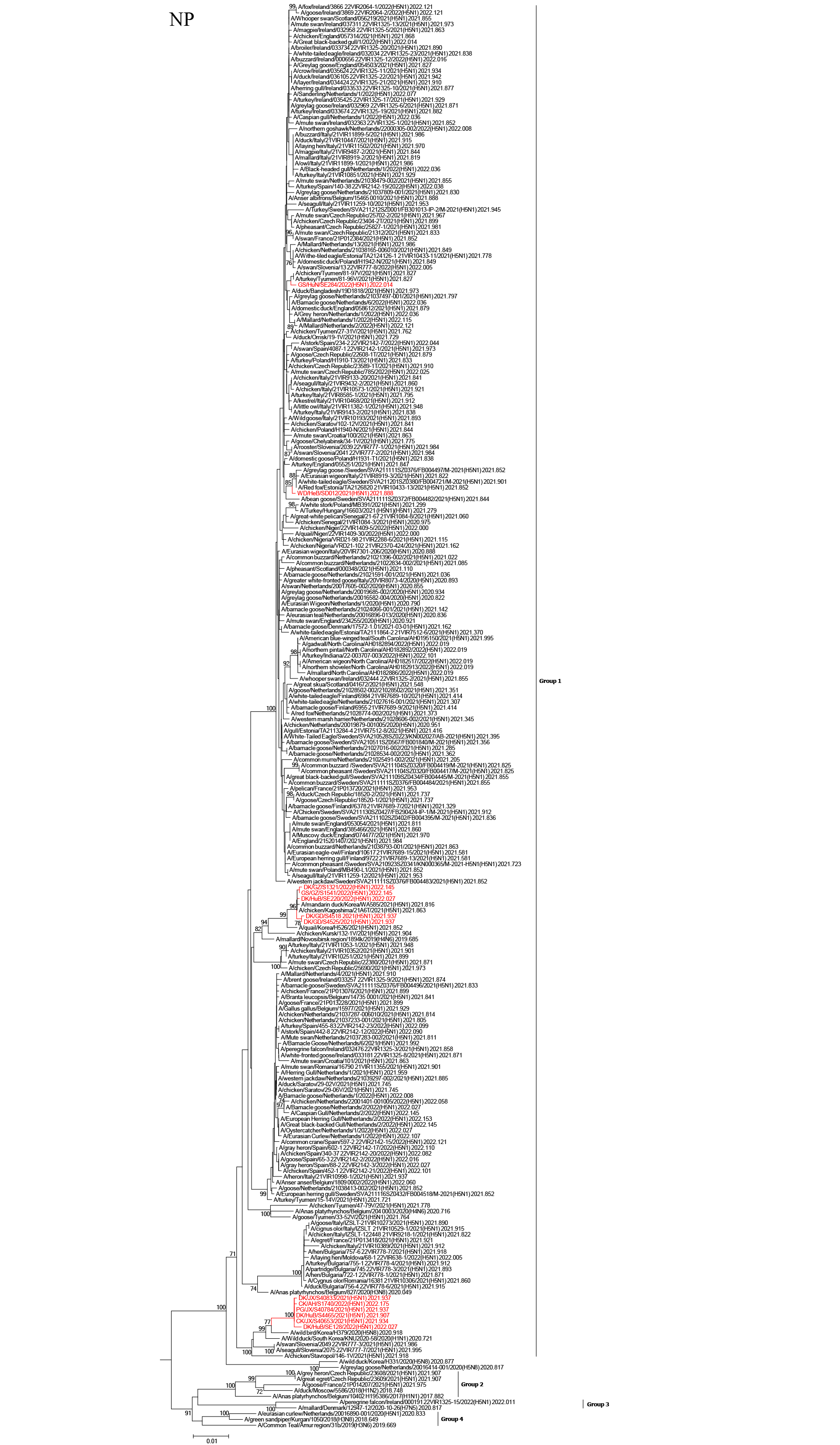

Supplement: Supplemental Material [file TEMI_A_2088407_SM9124.zip › Cui Fig S2e.tif]

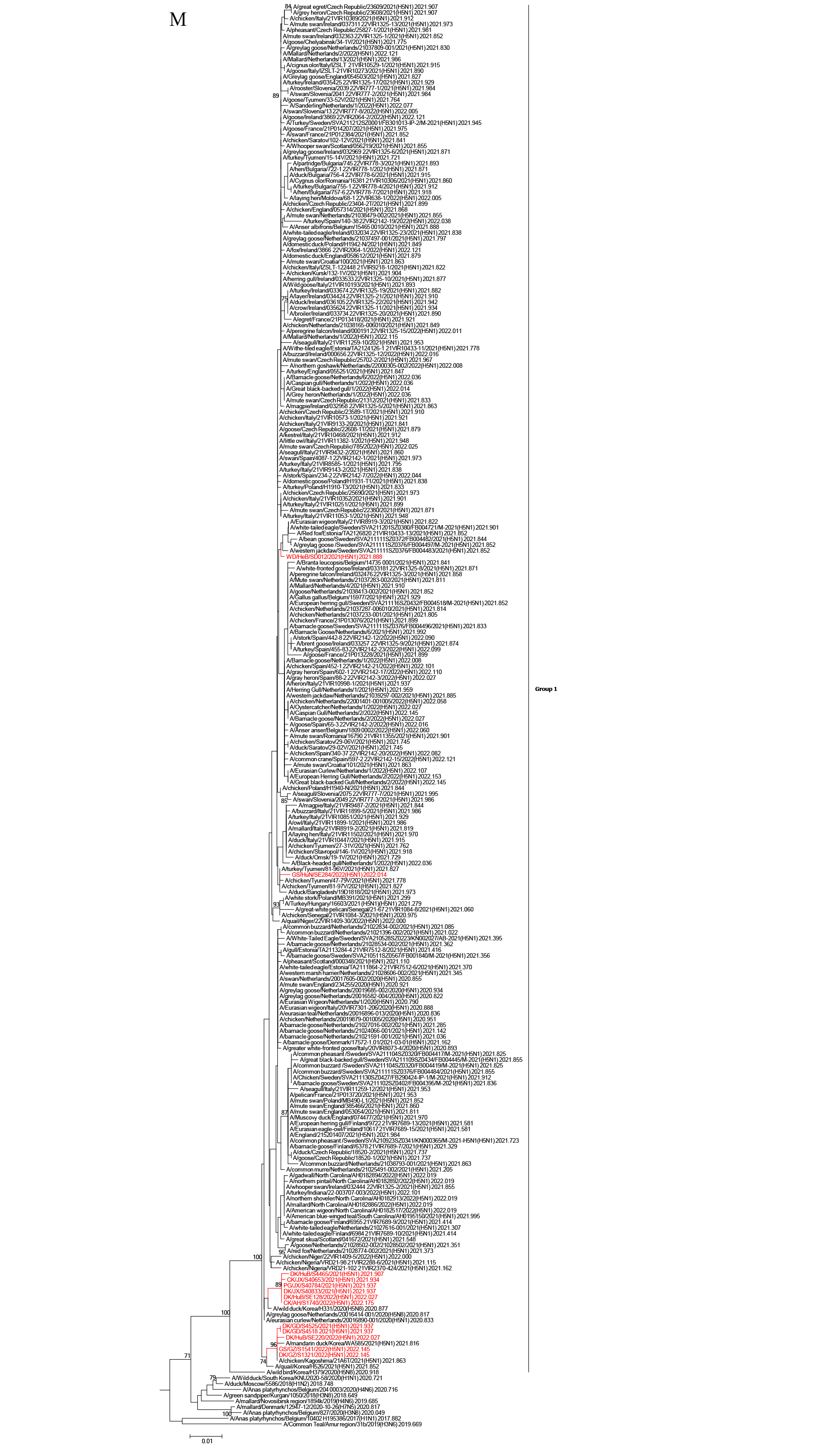

Supplement: Supplemental Material [file TEMI_A_2088407_SM9124.zip › Cui Fig S2f.tif]

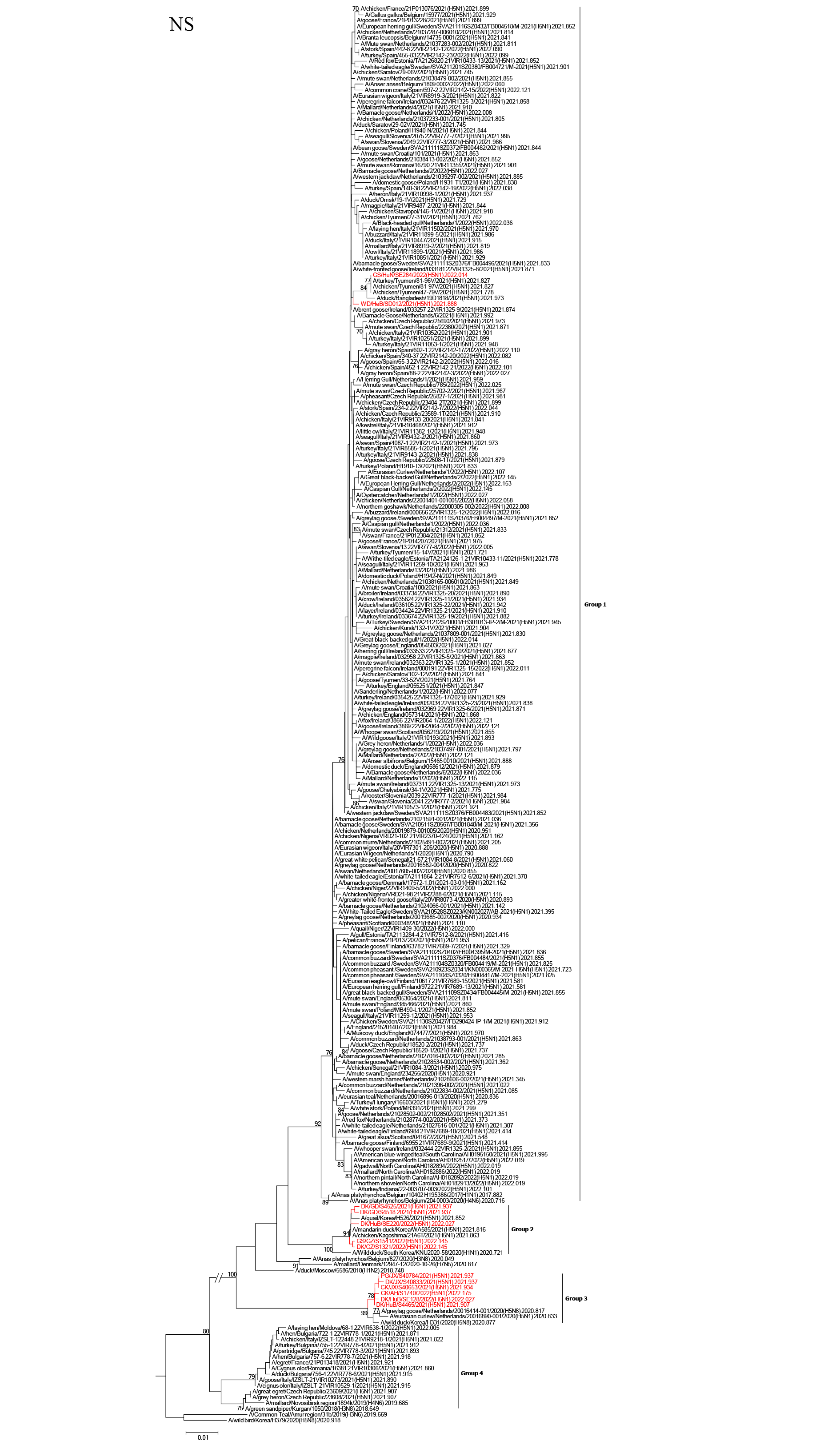

Supplement: Supplemental Material [file TEMI_A_2088407_SM9124.zip › Cui Fig S2g.tif]

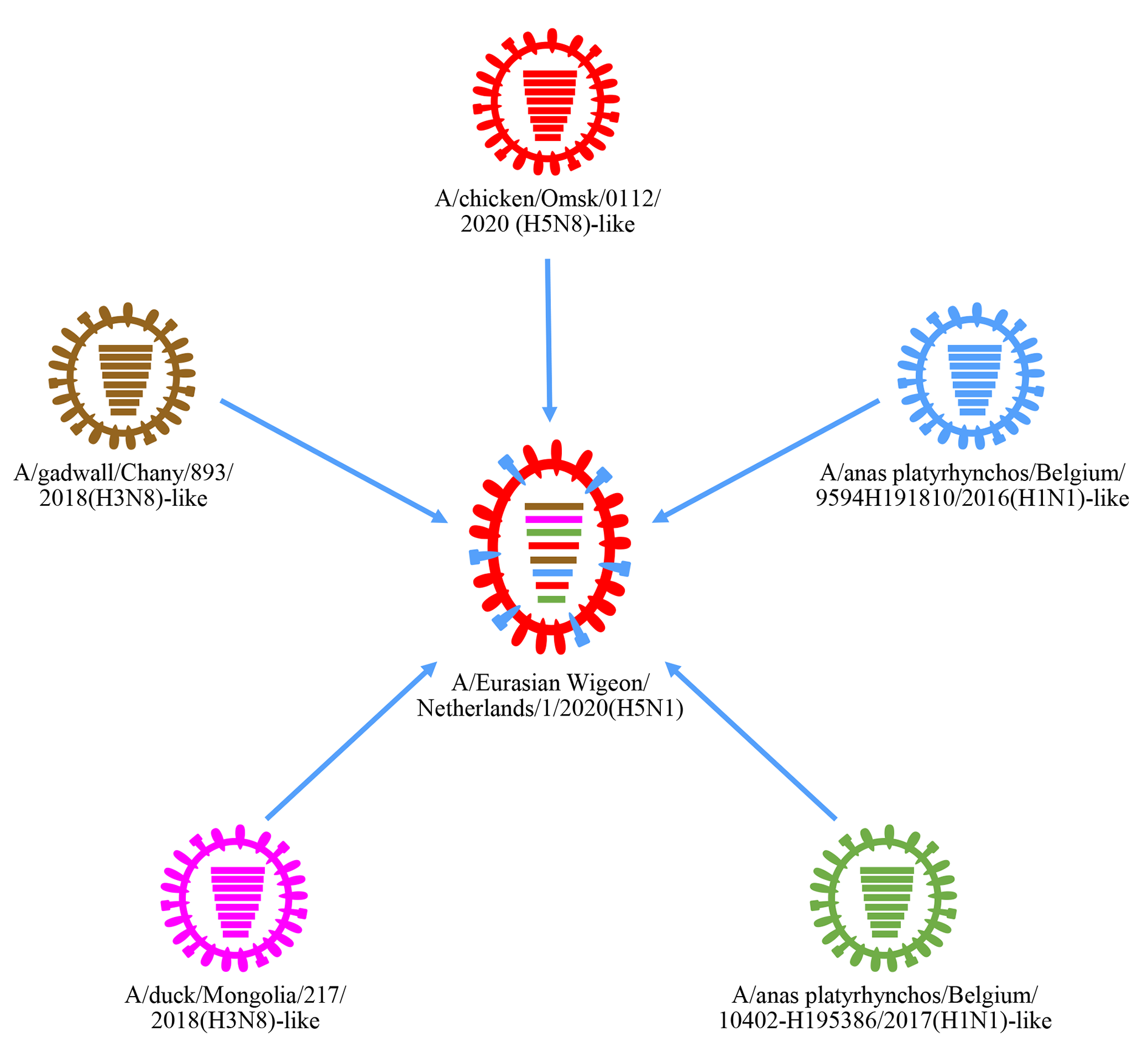

Supplement: Supplemental Material [file TEMI_A_2088407_SM9124.zip › Cui Fig S3.tif]
